# Seventy-five years of service: an overview of the College of American Pathologists’ proficiency testing program in histocompatibility and identity testing

**DOI:** 10.3389/fgene.2023.1331169

**Published:** 2023-12-19

**Authors:** H. Cliff Sullivan, Manish J. Gandhi, Sujata Gaitonde, Ramya Narasimhan, Ketevan Gendzekhadze, Soumya Pandey, Rhonda K. Roby, George C. Maha, Harmeet Kaur, Jennifer J. Schiller, Julie McDowell, Maria Smith, Chang Liu, Gerald P. Morris

**Affiliations:** ^1^ Department of Pathology and Laboratory Medicine, Emory University, Atlanta, GA, United States; ^2^ Mayo Clinic, Department of Laboratory Medicine and Pathology, Rochester, MN, United States; ^3^ Department of Pathology, University of Illinois Chicago, Chicago, IL, United States; ^4^ Boston University Medical Center, Department of Pathology and Laboratory Medicine, Boston, MA, United States; ^5^ City of Hope Medical Center, Duarte, CA, United States; ^6^ Department of Pathology, University of Arkansas for Medical Sciences, Little Rock, AR, United States; ^7^ Alameda County Sheriff’s Office Crime Laboratory, Oakland, CA, United States; ^8^ Retired, Chapel Hill, NC, United States; ^9^ Cuyahoga County Regional Forensic Science Lab, Cleveland, OH, United States; ^10^ Versiti Wisconsin Inc., Milwaukee, WI, United States; ^11^ College of American Pathologist (CAP), Chicago, IL, United States; ^12^ Washington University in St. Louis, Department of Pathology and Immunology, Saint Louis, MO, United States; ^13^ Department of Pathology, Univeristy of California San Diego, San Diego, CA, United States

**Keywords:** College of American Pathologists (CAP), proficiency testing, histocompatibility and identity testing committee, HLA antibody testing, HLA molecular typing, monitoring engraftment, disease association, HLA-B27

## Abstract

The Histocompatibility and Identity Testing Committee offers an overview of the College of American Pathologists’ (CAP) Proficiency Testing (PT) program, commemorating its significant 75th anniversary in 2024. The CAP PT program has undergone significant growth and evolution over the years, ultimately achieving Centers for Medicare and Medicaid Services approval. In 1979, CAP’s partnership with the American Association for Clinical Histocompatibility Testing marked a pivotal moment, leading to the creation of the first proficiency testing survey in 1980. This laid the foundation for various PT programs managed by the CAP Histocompatibility and Identity Testing Committee, including HLA antibody testing, HLA molecular typing, engraftment monitoring, parentage/relationship testing, HLA disease associations and drug risk, and HLA-B27 typing. Each program’s distinctive considerations, grading methodologies, and future prospects are detailed here, highlighting the continual evolution of histocompatibility and identity testing PT to support emerging technologies and evolving laboratory practices in the field.

## 1 Introduction

The CAP’s proficiency testing (PT) program will celebrate its 75th anniversary in 2024. In 1946, founding CAP Board member F. William Sunderman, MD, partnered with a group of Pennsylvania-based pathologists to conduct a statewide survey to evaluate the accuracy of some common chemical measurements. Following this initial survey, the CAP Standards Committee submitted a proposal to the Board of Governors in November 1947 to distribute a national survey of up to approximately 200 laboratories to assess the accuracy of laboratory determinations in anticipation of later distributing standards for calibration and equipment methods. By 1949, the program had expanded to 650 participants per mailing, with some 500 participants returning their results. Samples provided for analysis included water-based solutions of glucose, urea, chloride, and calcium, and a chloroform-based solution of cholesterol, each supplied at two levels.

In 1961, the CAP Board of Governors committed to expand the PT program “to develop and maintain the highest possible technical standards in the field of clinical pathology.” Surveys were no longer restricted to CAP members but were open to all laboratories. The CAP’s PT program was available as a Centers for Medicare and Medicaid Services (CMS) approved PT program when the Clinical Laboratory Improvement Amendments (CLIA) PT regulations were implemented in 1994 and has maintained approval every year since this implementation. The CAP continues to be a deemed organization by CMS and is in compliance with associated regulations (e.g., Title 42 of the Code of Federal Regulations Part 493). Additionally, the PT program is accredited by the American National Standards Institute (ANSI) National Accreditation Board (ANAB) and continues to be a distinguished laboratory quality improvement program designed and evaluated by panels of diverse experts from around the country, including the expertise from members of the CAP’s 28 scientific committees.

Just over 10 years later, the CAP partnered with the American Association for Clinical Histocompatibility Testing (AACHT; name changed to American Society for Histocompatibility and Immunogenetics in 1984) to form the AACHT/CAP Joint Committee for Histocompatibility Testing in 1979. The joint program produced its first Histocompatibility Survey in 1980, the same year CAP began accrediting Clinical Histocompatibility Laboratories. As of 2003, all CAP Histocompatibility surveys have been produced independently by the CAP. The CAP Histocompatibility and Identity Testing Committee (HITC), formed of members with expertise in histocompatibility and immunogenetics, designs the scope and focus of PT challenges and reviews all PT results prior to reporting to costumers. This insures the complexities of histocompatibility testing are accounted for when analyzing the results. The following sections detail the different PT programs that fall under the purview of the HITC. Each section is subdivided into four subsections, including an introduction to the PT, unique considerations, grading, and future prospects specifically related to each section’s content.

## 2 MX: HLA crossmatching, antibody screen, and antibody identification proficiency testing

### 2.1 Introduction to proficiency testing for anti-HLA antibodies

Antibodies against allogeneic human leukocyte antigen (HLA) molecules can cause rejection of solid organ allografts, platelet transfusion refractoriness, and delayed/non-engraftment in hematopoietic stem cell transplantation (Anasetti et al., 1989; Trial to Reduce Alloimmunization to Platelets Study Group, 1997; Girnita et al., 2005; Smith et al., 2011; Wiebe et al., 2012). Thus, accurate detection and characterization of anti-HLA antibodies is essential to support clinical transplantation. While a thorough description of the testing methods used and their strengths and limitations is beyond the scope of this current discussion, understanding the multiple assays used for clinical detection and characterization of anti-HLA antibodies is essential to identifying and meeting PT needs for histocompatibility laboratories. Crossmatch testing, both complement-dependent cytotoxic crossmatch (CDC) and the flow cytometry crossmatch (FCXM), directly test patient serum reactivity against cells from a given donor (de Moraes et al., 2019). This is highly informative for evaluating reactivity for a potential transplant pair but does not enable prospective assessment of anti-HLA antibody status before donor specimens become available. Antibody screening tests, currently formatted as flow cytometry-based immunoassays, directly determine the absence or presence of anti-HLA antibodies and estimate the breadth of reactivity against allogeneic HLA molecules, but may not identify the antigenic specificities of these antibodies (Bray et al., 2004). Information about specific reactivities of anti-HLA antibodies can be obtained by single-antigen bead (SAB) flow cytometry-based immunoassays, which enable precise identification of the antigenic specificities of anti-HLA antibodies present. More recently, SABs have been modified to use complement proteins such as C1q or C3d as detection reagents with a goal of differentiating “high risk” anti-HLA antibodies (though a physiologic basis for distinction based on this testing remains debated) (Lee et al., 2018). The CAP MX PT program groups these assays according to shared performance and function (Table 1).

**TABLE 1:** Methodologies tested by MX PT.

MX PT category	Testing methodologies supported	Test results reported
Antibody reactivity	Mixed antigen beads, PRA screening beads, SAB, PRA/SAB combination, SAB-C1q	Present/Absent
Antibody specificity	SAB, SAB-C1q	HLA specificities detected, in descending order of assay output
Crossmatch	CDC, anti-human globulin-augmented CDC, FCXM	Positive/Negative

^a^ PRA, panel-reactive antibody; SAB, single-antigen bead assay; SAB-C1q, C1q-binding SAB; CDC, complement-dependent cytotoxic crossmatch; FCXM, flow cytometry crossmatch.

The CAP MX survey provides comprehensive PT for anti-HLA antibody testing. MX survey challenges ship three times per year, with each shipment containing 4 samples (0.4 mL recalcified plasma) to be tested. Anti-HLA antibody testing can be performed using 6 screening and/or SAB assays and/or complement-binding-SAB assays (Table 1). Each shipment also contains 2 donor cell populations (>6 × 10^6^ peripheral blood lymphocytes) for cellular crossmatch testing. All samples are shipped at ambient temperature. Donor cell HLA genotyping information is also provided to enable laboratories to interpret crossmatch results as per routine clinical practice.

### 2.2 Unique considerations for proficiency testing for anti-HLA antibody testing

The genetic and phenotypic diversity of HLA is the most obvious challenge for developing and maintaining a robust PT program for anti-HLA antibody testing. While it is theoretically possible to cover each of the approximately 165 well-characterized HLA serologic reactivities (Holdsworth et al., 2009; Marsh et al., 2010) in the yearly PT samples, it is highly impractical and may not reflect the most clinically-relevant reactivities assessed in laboratories. Thus, care is taken to ensure that a representative range of potential antibody reactivities against HLA are assessed across PT samples within a year.

Another significant consideration for anti-HLA antibody testing is that most laboratories utilize multiple methods, either in isolation or in aggregate depending on the clinical context. Supporting multiple testing methodologies requires not only sufficient sample for different tests but also distinguishing how various methodologies can produce varying results on the same sample. For example, an antibody may be detectable by a screening assay and SAB, but not necessarily detectable by less analytically sensitive methods such as SAB-complement, CDC, or FCXM (Tait, 2016). However, this seeming discrepancy does not necessarily represent a failure of any of the methods to produce a “correct” result, but rather reflects the different clinical information provided by each assay. Thus, it is essential to grade PT results within similar methodology, or peer group, independently.

Finally, a significant challenge for grading PT results, even within peer groups, for anti-HLA antibody testing exists due to the diverse approaches that laboratories use to test and interpret anti-HLA antibody data. Anti-HLA antibody testing platforms, particularly SAB, have several known limitations to their performance including complement interference, non-specific reactivity, saturation, and non-linearity (Sullivan et al., 2017; Abraha et al., 2022). Although all laboratories are expected to have procedures in place to address these issues (Tait et al., 2013), there is no consensus or adoption of universal approaches which can affect the output of these assays. This may initially not seem to be a critical limitation for qualitative assays measuring anti-HLA antibodies, however, the raw output of these assays is semi-quantitative and laboratories establish their own policies for interpretation of these results (Liu et al., 2012; Sullivan et al., 2017). These interpretive approaches can be more or less stringent depending on the laboratory’s methods and the clinical needs (i.e., laboratories performing testing for hematopoietic stem cell transplantation (HSCT)) programs may use higher cutoff values for identifying potentially pathologic anti-HLA antibodies as it has been demonstrated that low-level antibodies may not present significant immunologic risk (Gladstone and Bettinotti, 2017). While this variability in the detection and characterization of anti-HLA antibodies may be clinically appropriate, it can create significant challenges when attempting to determine consensus results for PT samples.

### 2.3 Grading of proficiency testing for anti-HLA antibody testing

MX results are graded by consensus within each peer group, defined as the laboratories using a specific methodology to test the PT category. Antibody screening and crossmatch test results are reported as present/absent and positive/negative respectively. Antibody specificity results are reported as the specificities identified, listed in decreasing order of assay output. MX result reporting has 20 fields for listing antibody specificities for a given sample by a given method, with the ability to list additional specificities via free text entry. This approach of ranked listing is designed to minimize the effects of variation in interpretive approaches (more conservative interpretations are not necessarily penalized by more liberal interpretations of results, both of which may be clinically appropriate). This enables a reasonable approach to ensuring laboratories are detecting and characterizing the most clinically significant anti-HLA antibodies while preserving autonomy in medical practice. Results with 90% agreement within the peer group are determined as reaching consensus.

### 2.4 Future of proficiency testing for anti-HLA antibody testing

A practical challenge for anti-HLA antibody testing PT is the need to grade results in a way that ensures accuracy of the results obtained by individual laboratories, but enables flexibility in clinical practice. The degree of technical variation between methods, combined with the diversity in clinical practice, precludes approaches to PT that would be proscriptive. CAP HITC has focused on ensuring the reporting of meaningful qualitative data by participating laboratories (such as inclusive listing of identified anti-HLA antibody specificities) while avoiding quantitative data that may be imprecise and prone to misinterpretation (such as mean fluorescence intensity (MFI) data for SAB, which is known to vary widely between laboratories). However, the scale of potential diversity of PT challenge responses presents a challenge for data entry by PT participants as well as data collection and analysis by CAP. Efforts are continuing to find technical approaches to PT result reporting that are both intuitive and useful. Additionally, as new technologies are developed that may enable truly quantitative immunoassay output, HITC will need to evaluate the potential for quantitative as well as qualitative assessment of PT results.

Related to the challenge of providing PT that is clinically relevant across the spectrum of technical and clinical practice in transplantation, the HITC committee will need to address the increasing use of virtual crossmatch analysis. The practice of virtual crossmatch analysis, (Jackson, 2014), or determination of immunologic compatibility using donor HLA genotyping and recipient anti-HLA antibody testing data, is ever more common, driven primarily by changes in deceased donor organ allocation practices that expand access to donors outside of the recipient’s locality. While virtual crossmatch analysis is conceptually straightforward, intricacies related to anti-HLA antibody interpretation and differing clinical practices between transplant programs preclude universal approaches to PT grading of anti-HLA antibody interpretation and virtual crossmatch analysis. To date, CAP MX has assessed clinical interpretation of anti-HLA antibody testing results via informational (ungraded) written challenges, with the goal of providing information to MX participants regarding their clinical practice as compared to their peer group. The increasing prevalence and importance of virtual crossmatch analysis will continue to push the HITC committee to find new and improved approaches for assessing this in the MX survey.

## 3 DML: HLA molecular typing proficiency testing

### 3.1 Introduction to proficiency testing for HLA molecular typing

Clinical HLA typing can be performed at different levels of resolution, depending on the clinical application. Solid organ transplantation and transfusion support typically require identification of the serologic reactivity of the HLA molecules present (focused on a relatively limited set of polymorphisms on the surface-exposed elements of the HLA molecules) while HSCT requires determination of the specific HLA allele genotypes based on at least the regions encoding the antigen recognition domains (Exons 2 and 3 of HLA class I genes and Exon 2 of HLA class II genes), with the capability of differentiating common null alleles. Both “low-resolution” serologic split-level and “high-resolution” allele-level HLA typing are performed by DNA analysis methods. To support molecular typing of HLA, the CAP provides the DML PT survey which challenges the laboratory on their ability to provide accurate HLA genotyping results to the same level resolution at which the lab provides results to physicians for clinical decision making and patient care. The DML survey is shipped to participants twice per year (surveys DML-A and DML-B) and each sample containing 2 mL of whole blood in Citrate Phosphate Dextrose (CPD) or CPD-adenine (CPD-A) anticoagulant for 5 specimens in each shipment. Participants are expected to isolate DNA from each specimen using the lab’s routine DNA isolation procedure and perform molecular typing for each including all HLA genes tested in the lab. Laboratories participating in any given PT program are graded for their success in achieving results which are identical to or concordant with the majority of participants (based on 90% consensus).

The DML survey includes PT assessment for HLA-A, -B, -C, -DRB1, -DRB3/4/5, -DQA1, -DQB1, -DPA1, and -DPB1 genes. Each participating lab submits results for each specimen in the shipment and for each level of HLA typing performed. Reporting levels include generic typing (low-resolution 1^st^-field typing), Bw4 and Bw6 for each B-locus typing result, serologic equivalents associated with each generic typing result (if different from the first field molecular type and in concordance with United Network for Organ Sharing [UNOS] solid organ transplant patient requirements), and high-resolution 2^nd^-field typing, which meets HLA community standards as well as National Marrow Donor Program (NMDP) requirements for HSCT patients and donors.

Along with each molecular typing result submission, participants submit the specific typing methodologies and techniques used. The Participant Summary (PS) is published with this information included. Current categories of typing methodologies include real time qPCR, reverse sequence-specific oligonucleotide probe (SSOP), forward SSOP, sequence-specific primer (SSP), next-generation sequencing (NGS), and Sanger sequencing (Dunn, 2011). For each HLA locus, all typing results reported by participants, the frequencies, and the associated PT grade are also summarized in the PS to aid participants in understanding how their results compare to all other survey participants.

### 3.2 Unique considerations for proficiency testing for HLA molecular typing

PT of HLA molecular typing presents obstacles stemming from the intricate biological complexity and extensive genetic diversity of HLA genes, along with the technical intricacies of molecular testing methodologies. Biologically, HLA genes exhibit remarkably high polymorphism, boasting over 30,000 protein-coding variant alleles (https://www.ebi.ac.uk/ipd/imgt/hla/about/statistics/) (Holdsworth et al., 2009; Marsh et al., 2010), hindering the creation of comprehensive PT samples covering all variations. Additionally, the presence of sequence similarities among many HLA alleles results in typing ambiguities that require further testing and expertise to resolve. Analyzing the vast array of alleles at each locus and managing ambiguities becomes challenging when striving to reach a consensus.

Compounding the biological intricacies are the technical aspects of HLA typing, which encompass a multitude of methodologies and technologies employed in various laboratories, such as SSP, SSOP, Sanger sequencing, and NGS (Dunn, 2011; De Santis et al., 2013). Each method bears its own advantages and limitations, producing distinct levels of resolution, ranging from low-, intermediate-, and high-resolution typings. For example, intermediate-resolution may result in a long list (“string”) of possible alleles, whereas high-resolution typing can generally resolve the allele to two fields (e.g., HLA-A*02:01). These variations in methodologies, reagents, and reports across different laboratories make it challenging to compare and harmonize results. Furthermore, the analysis of complex molecular data and interpretation of HLA typing outcomes necessitate bioinformatics expertise, but PT software limitations mandate manual reviews, prolonging the consensus attainment and reporting processes.

To address the challenges inherent in molecular HLA typing and reporting, the CAP HITC continuously seeks improvement and implements measures to standardize data collection and grading. As of 2023, the committee has eliminated free text entry for reporting HLA alleles, requiring participants to input results into designated fields: one-field for generic typing and two- or three-fields for higher resolution typing. To circumvent the reporting of extensive strings of less common alleles that remain indeterminate but have uncertain clinical relevance, the data entry result form permits the selection of G (gene) and P (protein) groups. This grouping system diminishes the number of individual alleles or allele combinations reported while still preserving salient genetic diversity. Moreover, comprehensive DML kit instructions are included with examples defining criteria for high-resolution typing reporting, facilitating comprehension, and ensuring standardization. For instance, participants are reminded to report according to WHO-defined nomenclature (Marsh et al., 2010), exemplified by the following instruction: “If the consensus result is A*02:01:01G, then reporting A02:01G (incorrect nomenclature and not a WHO defined G group) or A*02:01:02G (also not a WHO defined G group) will be graded Unacceptable.”

By using these measures, HITC aims to enhance standardization, improve accuracy, streamline reporting, and expedite data analysis for HLA molecular typing PT. Collectively, these efforts contribute to the overarching goal of fostering consistency and precision in HLA molecular typing across laboratories.

### 3.3 Grading proficiency testing for HLA molecular typing

Grading of participant submissions for PT assessment is often more complicated than it may seem. Although HITC strives to provide a simple and straightforward interface for submission of results, differences in laboratory practice with respect to the level of typing resolution and even misinterpretation on how to appropriately enter PT results for the assessment create challenges. All results submitted in the result entry form for the “1st Type” and “2nd Type” (corresponding to each of the 2 alleles expected in each sample for each locus) are most readily summarized and graded based on consensus. However, many laboratories additionally enter free-text comments along with each locus submission including information on additional alleles, which they have not ruled out using their typing methodology or reporting procedure. These comments need to be individually reviewed by the committee to assess whether or not their inclusion still leads to an appropriate high-resolution HLA typing result with respect to G groups and P groups as required and defined for high-resolution typing requirements per the most-recent catalog version of Common, Intermediate and Well-Defined (CIWD) HLA alleles and the resolution of common null alleles which may be present in G groups being reported (Hurley et al., 2020).

As a PT participant, some of the additional grading challenges routinely encountered by the committee, which the laboratory should be keenly aware of, include the submission of more than two antigenic groups for any locus, or the submission of results utilizing incorrect nomenclature which does not align with current WHO standards. An example of a generic typing submission which would receive a grade of Unacceptable is any entry in which more than two antigens are submitted for a single locus (when considering the comment field in addition to the DML 1st Type and 2nd Type entry fields). Examples of high resolution submissions which would receive a grade of Unacceptable include HLA-A*01:01G, as the correct nomenclature is HLA-A*01:01:01G (Acceptable), HLA-B*39:11P (Unacceptable) when no such P group exists, and HLA-DQB1*02:02G (Unacceptable) where the correct nomenclature is HLA-DQB1*02:01:01G (Acceptable) and the allele identified by consensus is HLA-DQB1*02:02 (Acceptable).

Based on the above assessments of participant result entries, nomenclature, and anything additional entered into the comments field on the data submission form, results that achieve 90% participant consensus (based on a combination of Good and Acceptable grade categories) are formally graded for each participant. Results that do not achieve such consensus are reported as Ungraded. Table 2 provides examples of the grading categories while Table 3 provides the consequences of receiving unacceptable PT results.

**TABLE 2:** HLA high resolution genotyping grading categories.

Molecular typing result	Grading category
Unambiguous results consistent with consensus	Good
Ambiguous results consistent with the consensus and including alternate alleles (provided in the Comments) which are in the same G/P group	Acceptable
Ambiguous results consistent with the consensus and including well-documented or rare alternate alleles outside of the same G/P group	Acceptable
Ambiguous results consistent with the consensus and including common or intermediate alternate alleles outside of the same G/P group	Unacceptable
Results inconsistent with the consensus (incorrect alleles identified and/or submitted using incorrect nomenclature)	Unacceptable

**TABLE 3:** Consequences of receiving unacceptable PT results.

PT performance	Definition	Impact on testing
Unsatisfactory	Receiving an unacceptable result for a given analyte or test during a PT event	Can continue testing until the next PT event
Unsuccessful	Receiving unsatisfactory PT performance on 2 consecutive or 2 of 3 PT events	Can continue testing until the next PT event
Critical	Receiving unsatisfactory PT performance on 3 consecutive or 3 of 4 PT events	Can continue testing until the next PT event. Laboratory will be warned they are at risk of a cease testing directive
Repeat Critical	Receiving unsatisfactory PT performance on 4 consecutive or 4 of 5 PT events	May be directed to cease testing on given analyte or test until reinstatement requirements have been met

^a^ These consequences are applicable to all CAP PT programs.

### 3.4 Future of proficiency testing for HLA molecular typing

The continuous advancement of HLA molecular typing poses future challenges for PT. The ongoing discovery of novel and rare HLA alleles leads to an expanding pool of variants, necessitating continual updates to PT samples and data entry forms for comprehensive coverage. The emergence of new NGS technologies facilitates interrogation of HLA gene sequences including introns and non-coding regulatory sequences, and the clinical relevance of this increased level of genotyping is actively under investigation (Mayor et al., 2019; Mayor et al., 2021). As some clinical laboratories will undoubtedly begin to report this additional genotype information for clinical use, this will require PT that supports this level of testing. Additionally, evolving regulations and accreditation requirements may impact PT grading procedures.

To address these challenges proactively, the HITC must remain dynamic and responsive to HLA molecular typing advancements. Regular updates to the data entry form will ensure alignment with the evolving HLA landscape, accommodating new alleles and high-throughput sequencing technologies. Along with the discovery of new alleles comes the assignment of appropriate serological equivalents. As the HLA community reaches a consensus on serological equivalents for these new alleles, the committee will need to update PT reporting accordingly. Furthermore, the committee anticipates a transition to universal high-resolution typing, making generic typing PT potentially obsolete.

## 4 ME: monitoring engraftment proficiency testing

### 4.1 Introduction to proficiency testing for monitoring engraftment

Chimerism testing quantifies the relative amounts of donor and recipient-derived hematopoietic cells based on measurement of distinguishable genetic polymorphisms. Chimerism testing is most commonly used to evaluate donor engraftment after HSCT. Underlying malignant or non-malignant disease, patient conditioning regimen, graft cellular content, graft manipulation, and posttransplant treatment for Graft-versus-host disease (GVHD) and infections, affect the chimerism kinetics and should be considered in interpretation. Engraftment can be associated with relapse and GVHD and must be performed before donor lymphocyte infusion (DLI) or consecutive transplants. A panel of experts has agreed on the definitions of cellular recovery, graft failure, poor graft function, secondary graft failure, and which chimerism test should be used to diagnose these complications and interpret them accordingly (Kharfan-Dabaja et al., 2021). Chimerism testing can be performed on peripheral blood, bone marrow, and specific cellular subsets, including myeloid or lymphoid compartments. For purposes of the CAP Monitoring Engraftment (ME) survey, “full” chimera is present if donor DNA is >95% for both myeloid and lymphoid lineages, “mixed or partial” if the result is 5%–95%, and “absent” if donor DNA is less than 5%. The panel recommended chimerism testing time points and type of subsets to analyze according to disease type and conditioning regimen. Besides the HSCT setting, chimerism testing is critical to detect rare but high-risk occurrences of GVHD associated with transfusion and liver transplantation.

The ME survey is conducted twice per year. For each survey, samples are mixed to form 5 admixtures from unique pairs, referred to as ‘A’ donor and ‘B’ recipient. Thus, over the course of a year, the survey assesses a total of 10 admixtures and 4 individual blood samples (2 pairs). These samples, each containing 0.5 mL and preserved in either CPD or CPT-A anticoagulant, are kept at room temperature.

### 4.2 Unique consideration for proficiency testing for monitoring engraftment

Multiple methods for chimerism analysis exist, with the analytical sensitivity and specificity determined by 2 main factors: 1) selection of informative markers to distinguish the recipient from the donor; and 2) detection method. Historically, blood groups and gender-specific markers were tested using agglutination, flow cytometry, or conventional cytogenetics methodology, but today most of the clinical laboratories are using molecular assays. Molecular markers vary from SNP (single nucleotide polymorphism), indels (insertion/deletion), VNTR (variable number of tandem repeats), STR (short tandem repeats), or a combination of them. Multiple quantification methods include FISH (fluorescence *in situ* hybridization), RFLP (restriction fragment length polymorphism), DNA fragment analysis sequencing, Real-time PCR, digital PCR, and NGS. Though many methods have been developed, most participants (115 and 104 from 135 participants from ME-A, 2023) are using commercial kits and capillary electrophoresis STR analysis (respectively) as the detection method.

### 4.3 Grading proficiency testing for monitoring engraftment

The participants receive acceptable grades if the final donor (“A”) proportion (percentage) reported is within the range of consensus, which is defined as mean ±3 standard deviations (SDs). The PS includes lower and upper limits and SDI (Standard deviation index) as additional information for participants to evaluate their performance. The result is the average percentage of all informative markers and can be inaccurate if only a few informative markers are used. Unfortunately, the selection of too few markers continues to be an issue, 28/120 laboratories used only 1-2 informative markers for calculation (e.g., ME-A, 2023).

**FIGURE 1:**
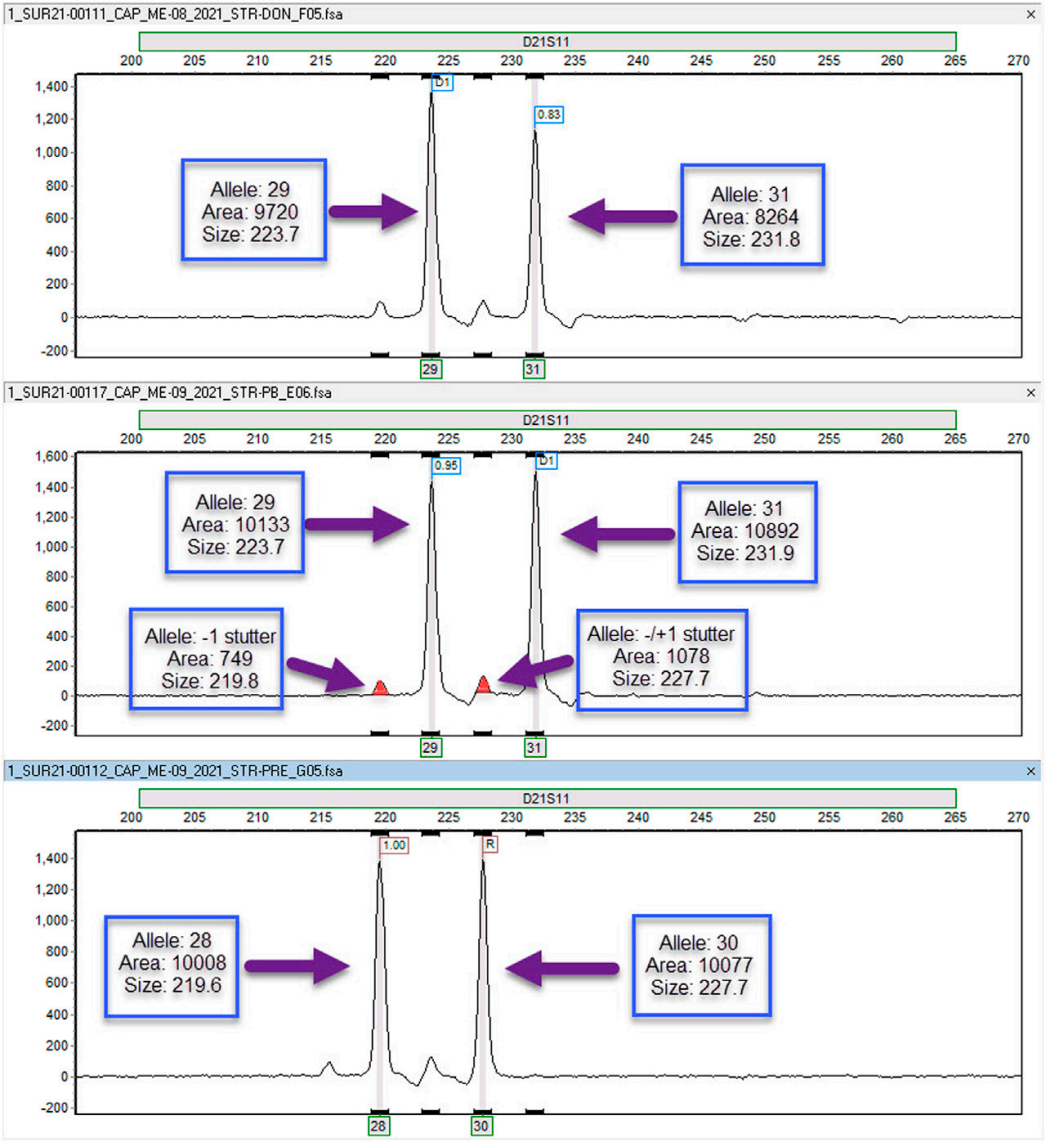
Electropherograms demonstrating stutter Electropherograms for the D21S11 short tandem repeat (STR) marker. The top panel is the electropherogram for the donor while the bottom panel is the electropherogram for the recipient pre-transplantation. The middle panel is the electropherogram for the recipient post-transplantation (allele, area = peak area, size = allele size). The post-transplant sample demonstrates that the recipient alleles (allele 28 and allele 30) are in the stutter of the donor alleles (allele 29 and allele 31). The presence of stutter peaks (represented by the smaller solid peaks) introduces complexity when attempting to interpret the percentage of donor and recipient contributions. This challenge arises due to the overlapping nature of recipient and donor markers. Typically, markers exhibiting stutter peaks are not considered informative for determining percent chimerism. Nevertheless, it is possible to utilize these markers if specific calculations are employed to accommodate for the average percent stutter associated with the particular marker in question.

ME evaluation is also challenging when the sample only contains component “A” or “B”, due to limitations in analytic sensitivity [STR sensitivity is around 1%–5% (Picard et al., 2023)]. Of note, a small number of laboratories use methods like NGS, real-time PCR or digital PCR, which can reach a limit of detection down to 0.01%–0.1% (Picard et al., 2023). Discrepant results are usually due to wrong interpretation of “stutter” bands, which are PCR errors due to strand slippage during primer extension (Levinson and Gutman, 1987), resulting in 1 less STR. Most of the time, markers with “stutter” bands should not be used as informative markers (Hancock et al., 2003). In a 2021 survey, seven markers were identified as having “stutter” bands (Sample ME-14, ME-B, 2021) and 3 electropherogram examples were provided (see Figure 1 for 1marker: D21S11) in the discussion to address multiple participants’ concerns for discrepant grading. Nonetheless, participants are expected to distinguish monotypic samples from admixtures.

### 4.4 Future of proficiency testing for monitoring engraftment

The current survey faces a persistent challenge with the limited availability of human blood samples and transplant scenarios. As we look ahead to future surveys, it will be imperative to explore potential solutions for this issue. For example, the detection of informative markers typically relies on pre-transplant blood samples. However, an alternative option lies in using DNA isolated from buccal brushes or hair, which is sometimes employed by clinical laboratories. It is worth noting that the quantity and quality of buccal DNA can be compromised, significantly impacting the amplification reaction. Therefore, it may be advisable to include buccal samples in future survey evaluations.

The source of PT samples should be a focal point in future surveys as well. Currently, the PT relies on samples obtained from healthy donors, which contrasts with the reality of clinical scenarios where samples are sourced from patients with hematologic malignancies. This discrepancy between PT samples and clinical samples deserves consideration. However, obtaining DNA from immunosuppressed transplant patients presents formidable challenges in terms of both quantity and quality, rendering it nearly impractical to incorporate such scenarios into the survey.

Chimerism testing also offers a valuable tool in cases involving multiple transplants. In these situations, chimerism testing can effectively distinguish the admixture of DNA from more than two distinct individuals. To accommodate this aspect, expanding the survey’s scope by including PT samples from two or more individual donors could prove beneficial. Additionally, chimerism testing finds relevance in identifying monozygotic twins, a potential factor in HSCT donor selection. In such cases, the patient and their identical twin lack informative markers, precluding engraftment chimerism assessment.

In terms of assay sensitivity, several emerging methodologies like NGS, real-time or digital PCR, with varying degrees of sensitivity, have been adopted by some laboratories, which can create evaluation challenges. In connection with the growing adoption of high-throughput technologies, the utilization of expanded sets of genetic markers is evident in distinguishing admixture genotypes. The integration of large panels of SNPs or markers, different from the predominantly employed STRs, should not necessarily impact the final determination of the proportion of admixture. However, it poses a challenge in furnishing participant data related to the incorporation and performance of individual markers in the PS. As the use of these high-throughput assays increases, HITC will discontinue the inclusion of this data in the PS in 2024.

The sensitivity of capillary electrophoresis STR analysis can be improved using cell subsets (Antin et al., 2001); 68% of respondents of a survey perform T cell chimerism where T cells are separated using CD3 magnetic beads and others also test the myeloid/granulocyte population (Clark et al., 2015; Blouin et al., 2021). Purity assessment by flow cytometry is recommended before DNA isolation. Cell purity is yet another parameter that could be evaluated by future surveys.

In summary, the ME survey is poised to evolve and adapt to the rapidly changing methodologies that have emerged in recent years. It will encompass not only various sample types but also diverse transplant scenarios to ensure its continued relevance and effectiveness.

## 5 PARF: parentage/relationship test—filter paper proficiency testing

### 5.1 Introduction to proficiency testing for parentage/relationship

The PARF (Parentage/Relationship Test—Filter Paper Proficiency Testing) survey was first offered jointly by the CAP and the American Association of Blood Banks (AABB, now the Association for the Advancement of Blood and Biotherapies) in 1993 as the PI survey, or Parentage Identity (Polesky et al., 1996). When the Parentage survey was introduced, the laboratory results reported by participants included red cell antigens, red cell enzymes, serum proteins, HLA, and DNA results. The early surveys’ DNA results were RFLP and later autosomal STRs. With the reported phenotypes, the calculated paternity index/likelihood ratio (LR) values were also submitted. As technology advanced, fewer participants reported non-DNA results, and RFLP results gradually dwindled until no laboratories currently report RFLP results. Presently, participants exclusively report standardized STR testing, which includes autosomal STR, Y-STR, and emerging X-STRs.

Laboratories accredited by the AABB are mandated to partake in PT, which assesses their capability to deliver accurate phenotyping results and analyses crucial for constructing relationship testing reports. Participants must report results for all loci and assays used in casework, along with the corresponding LR. The CAP is a PT provider accredited by the ANSI National Accreditation Board (ANAB) to the international standard ISO/IEC 17043:2010, as recommended by the Paternity Testing Commission of the International Society of Forensic Genetics (Morling et al., 2002). PARF meets this standard.

Triannually, four different biological specimens obtained from volunteers are distributed to participating relationship testing laboratories through the CAP PARF survey consisting of three mailings: PARF-A, PARF-B, and PARF-C. The biological specimens previously consisted of whole blood and/or buccal swabs. In recent years, the biological specimens mimic the majority of specimens received by relationship testing laboratories for paternity testing, which consist of mock buccal swabs and bloodstains on filter paper. All shipments consist of a mother, child, tested man #1 (alleged father #1), and tested man #2 (alleged father #2). For one shipment, the four samples include blood-stained filter cards for each biological specimen. For two shipments each year, they contain blood-stained filter paper for the mother and child specimens and mock buccal swabs for the two alleged fathers. The survey testing of these biological specimens aims to maintain standard paternity trios but includes two alleged fathers in each submitted case—one alleged father is included while the other is excluded. Notably, closely related alleged fathers, such as biological siblings or a father and son, have never been used as the two alleged fathers.

Also included in each PARF survey is a calculation challenge (also known as a paper challenge or a dry challenge). Most often, the paper challenge consists of a case scenario, phenotyping results, and frequencies for each allele at each locus, and a few questions for each participating laboratory to answer. For the paper challenge, the participants are asked to report the LR result for each locus as well as the combined LR. No biological specimens are distributed for the paper challenge. By providing the phenotyping results and the allele frequencies, the responses address reporting and not the laboratory work conducted, which removes any differences in reported phenotypes and any differences due to the frequencies in the database used.

### 5.2 Unique consideration for proficiency testing for parentage/relationship

The methods used and the nomenclature of reporting laboratory results have a high degree of standardization today. As the technology has advanced, so has the consistency in reporting. Nonetheless, typing discrepancies are encountered and must be taken into consideration:

1) The 9.3 allele of the HUMTH01 locus is often observed in reporting this locus due to its high frequencies in the commonly used populations. In the surveys offered through the 1990s, the resolution from the technology in use at that time for the 9.3 and the 10 alleles was not always precise enough for the reporting laboratories; the participants would pool these results into reporting a 9.3/10 result instead of a distinct 9.3 allele and a 10 allele. Today, with the one base pair resolution that is easily achieved with the different capillary electrophoretic instruments, reporting of the 9.3 at this locus is possible.
2) Rarely, consensus is not achieved due to the use of different commercially available kits; the kit manufacturers are known to have different primer sequences and different amplification efficiencies. Typically, there are two to three kit manufacturers that are used for the majority of the reporting laboratories. However, more often, differences are observed when a participating laboratory designs its own kit internally.
3) The most common reporting difference amongst the participants is the reporting of genotypes *versus* phenotypes. Reporting the phenotype is scientifically accurate. When a participant reports a homozygous genotype, *e.g.,* 13, 13, it is flagged with a footnote that the “participant is incorrectly reporting genotype rather than reporting phenotype when a single allele is visualized.” For a homozygous result, a grade of “good” is reported when the participant reports the correct one allele at the locus and a grade of ‘acceptable’ is reported when the participant reports the correct allele but has two alleles at the locus. The participants should report the phenotype rather than the genotype when only one allele is visualized using STR methods.

### 5.3 Grading proficiency testing for parentage/relationship

A grading scheme for the 3 levels of performance (good, acceptable, and unacceptable) was introduced in 1997 for responses reported by the participants (Allen et al., 2003). With a minimum of 10 participants reporting a result, that result would be graded if 9 participants reported the same result (Polesky et al., 1998). This grading evolved and for qualitative results, consensus is now established when at least 10 participants report results and at least 80% of those results are in agreement. When consensus is achieved, grading is provided at each locus. Grading is reported on both interpretation results (e.g., alleles) and numerical values (parentage index/likelihood ratios). STR results that do not reach the minimum number of participants or do not reach consensus are not graded. Further, the calculation paper challenge is not graded.

Over the many years of distributing this and versions of this survey, reporting differences have been observed and documented. In 2003, Allen et al. reported the percent of incorrect quantitative results for the LRs may be due to the limited number of allele frequency databases in use by participants (Allen et al., 2003). Further, Allen et al. proposed increasing the magnitude of the standard deviations used for grading to increase the range of acceptable responses and reducing the unacceptable response rate. Through 2023, quantitative results of the LRs have been graded after the outliers were removed with a mean based on the submitted responses and 3 standard deviations.

However, the committee recognizes that pseudoreplication (Hurlbert, 1984; Lazic, 2010) is possibly the cause of some of the unacceptable responses graded by the CAP Subcommittee and that most of these participants are likely accurately applying the correct LR calculations but possibly using a database very different than some of the commonly used databases. Hence, one to three databases used make up the majority of the responses and therefore, are not independent. In short, pseudoreplication can be an effect of replicates that are not statistically independent. Therefore, when the mean and standard deviation were calculated for the LRs, all of the responses were not independent since many participants used one of three databases. This leads to multiple observations from the same database, which in turn leads to dependencies that are skewed. Therefore, in 2023, the subcommittee decided not to grade some of the results by committee decision for PARF-B 2023 and PARF-C 2023; starting in 2024, the LRs will not be graded.

### 5.4 Future of proficiency testing for parentage/relationship

With the increasing use of the sex chromosomal testing of Y-STRs and X-STRs, it is anticipated that more participants will be reporting these results and thus, consensus may be achieved in some of these systems that have not been previously achieved. Further, with the increasing use of new technologies, such as NGS, there will be reporting strategies that must be considered and reporting of these results compared to the standard STR results today and those obtained from the different technologies. To address these challenges proactively, the CAP HITC must remain dynamic and responsive to molecular typing advancements. Regular updates to the data entry form will ensure alignment with the evolving landscape, accommodating new alleles and high-throughput sequencing technologies.

## 6 DADR: disease association and drug risk proficiency testing

### 6.1 Introduction to proficiency testing for HLA disease association and drug risk

In addition to utility for transplantation, HLA genotypes have significant associations with several disease states, most notably autoimmunity and adverse hypersensitivity drug reactions (Gough and Simmonds, 2007; Jeiziner et al., 2021). PT to support focused HLA typing for HLA disease association and drug risk (DADR) is provided by the CAP. Three 0.1 mL specimens, each containing 200 μg/mL of extracted human DNA are shipped in kits with specific instructions twice a year for each analyte. The specimens are intentionally sent at ambiently. Upon opening, the specimens are stable for 48 h when tightly capped and stored at room temperature. Unopened specimens are stable at room temperature for a duration of 30 days. All laboratories are asked to measure and adjust nucleic acid concentrations according to standard laboratory protocols prior to performing nucleic acid amplification procedures. Both surveys also contain paper (dry) challenges regarding the clinical relevance of HLA genotypes in non-transplant-related diseases for educational purposes.

Drug risk (DADR1) assesses the detection of HLA-A*31:01, HLA-B*13:01, HLA-B*15:02, HLA-B*57:01, and HLA-B*58:01. This survey challenges the laboratory to accurately identify the presence or absence of alleles associated with the adverse reactions to specific drugs such as carbamazepine-induced Stevens-Johnson syndrome, allopurinol-induced Stevens-Johnson syndrome, abacavir hypersensitivity, and dapsone hypersensitivity. Disease association (DADR2) tests the detection of the following alleles: HLA-A*29:01, HLA-A*29:02, HLA-DQA1*04:01, HLA-DQA1*05:01, HLA-DQB1*03:02, HLA-DQB1*06:02, HLA-DRB1*03:01, HLA-DRB1*03:02, HLA-DRB1*04:02, HLA-DRB1*04:03, HLA-DRB1*04:06, HLA-DRB1*08:02, HLA-DRB1*08:04, HLA-DRB1*14:04, HLA-DRB1*14:05, HLA-DRB1*14:08, HLA-DRB1*15:01, HLA-DRB1*15:02, DQA1*02, DQA1*03, DQA1*05, DQB1*02:01, and DQB1*02:02. This survey tests the laboratory ability to accurately recognize the presence or absence of alleles associated with disease states such as celiac disease (CD), narcolepsy (N), pemphigus vulgaris (PV), psoriasis (P), anti-glomerular basement membrane disease (ABM), birdshot retino-choroidopathy (BR), and idiopathic myopathy (IM). Table 4 presents examples of well-established HLA drug and disease associations for specific alleles.

**TABLE 4:** DADR analytes for disease association and drug risk.

DADR1		
Analyte	Associated drug or disease risk	References
HLA-A*31:01	carbamazepine hypersensitivity	NEJM 2011; 364:1134–1143 (McCormack et al., 2011)
HLA-B*13:01	dapsone-induced cutaneous adverse reactions	NEJM 2013; 369:1620–1628 (Zhang et al., 2013)
HLA-B*15:02	carbamazepine hypersensitivity	NEJM 2011; 364:1126–1133 (Chen et al., 2011)
HLA-B*57:01	abacavir hypersensitivity	NEJM 2008; 358:568–79 (Mallal et al., 2008)
HLA-B*58:01	allopurinol hypersensitivity	BMJ 2015; 351:h4848 (Ko et al., 2015)
HLA-A*29:01	birdshot retino-choroidopathy	Ocul Immunol Inflamm 2011; 19: 397–400 (Brezin et al., 2011)
HLA-A*29:02	birdshot retino-choroidopathy	Ocul Immunol Inflamm 2011; 19: 397–400 (Brezin et al., 2011)
**DADR2**		
**Analyte**	**Associated drug or disease risk**	**References**
HLA-DQB1*06:02	Narcolepsy	Immunol Res 2014; 58: 315–339 (Mignot, 2014)
HLA-DRB1*03:01	Sjӧgren’s syndrome	Autoimmun Rev 2012; 11: 281–287 (Cruz-Tapias et al., 2012)
HLA-DRB1*04:02	pemphigus vulgaris	Br J Dermatol 2012; 167: 768–777 (Yan et al., 2012)
HLA-DRB1*04:03	pemphigus vulgaris	Br J Dermatol 2012; 167: 768–777 (Yan et al., 2012)
HLA-DRB1*04:06	pemphigus vulgaris	Br J Dermatol 2012; 167: 768–777 (Yan et al., 2012)
HLA-DRB1*14:05	pemphigus vulgaris	Br J Dermatol 2012; 167: 768–777 (Yan et al., 2012)
HLA-DRB1*14:08	pemphigus vulgaris	Br J Dermatol 2012; 167: 768–777 (Yan et al., 2012)
HLA-DQA1*02	celiac disease	Hum Immunol 2020; 81: 59–64 (Choung et al., 2020)
HLA-DQA1*05	celiac disease	Hum Immunol 2020; 81: 59–64 (Choung et al., 2020)
HLA-DQB1*02:01	celiac disease	Hum Immunol 2020; 81: 59–64 (Choung et al., 2020)
HLA-DQB1*02:02	celiac disease	Hum Immunol 2020; 81: 59–64 (Choung et al., 2020)
HLA-DQB1*03:02	celiac disease	Hum Immunol 2020; 81: 59–64 (Choung et al., 2020)

### 6.2 Unique considerations for proficiency testing for HLA disease association and drug risk

This is a rather straightforward survey with good agreement among participants. The survey currently has DQB1*02:01 and DQB1*02:02 as separate analytes. They both belong to the same DQB1*02:01P and DQB1*02:01:01G group and are associated with celiac disease (Sciurti et al., 2018). Participants may not be able to differentiate between these alleles, depending upon the test methodology used, which may occasionally cause disprepant results.

### 6.3 Grading proficiency testing for HLA disease association and drug risk

The HLA typing methodologies used by participants include real-time PCR, NGS, Sanger sequencing, forward SSOP, reverse SSOP, and SSP in various combinations. Methods that do not fall under the listed methods can be entered under the “other test methodology category.” Results are simply recorded as present or absent. Performance grading for the DADR1 and DADR2 Surveys is based on 80% participant consensus and/or the intended response as established by the referee laboratory’s result. Survey results are not stratified by test methodology. There must be at least 10 laboratories in the peer group to report a result. The CAP uses exception reason codes for ungraded results. The laboratory must identify all analytes with an exception reason code, review, and document the acceptability of performance as outlined in the instructions and retain documentation of review for at least 2 years. The survey occasionally has educational dry challenge with multiple choice questions, which help keep participants’ knowledge up-to-date regarding HLA disease association and drug risk.

### 6.4 Future of proficiency testing for HLA disease association and drug risk

The field of pharmacogenomics and research in HLA disease associations is rapidly growing and new disease associations and HLA-drug risks are being identified. For example, the survey currently does not include an HLA-A*02:01 analyte, which is increasingly utilized as a requisite for cancer immunotherapies. As an example, the frontline treatment for unresectable or metastatic uveal melanomas is Tebentafusp, an immune-mobilizing monoclonal T cell receptor that has a high binding affinity for the melanoma-associated antigen gp100 presented by HLA-A*02:01 (Chen and Carvajal, 2022). Incorporating this analyte, along with others as they become integrated into routine clinical practice, is a significant undertaking essential for maintaining the relevance of this survey.

## 7 B27: HLA-B27 typing proficiency testing

### 7.1 Introduction to proficiency testing for HLA-B27 typing

Patient HLA-B27 screening for ankylosis spondylitis and associated spondyloarthropathies is performed by many US and international CAP accredited laboratories. Following CLIA guidelines, CAP provides PT material: 5 whole blood specimens twice a year. PT specimens are shipped at room temperature and stay stable for 3 days once opened and 7 days unopened. Participating laboratories are expected to test these specimens as if they were patient specimens and submit their results as HLA-B27 “present” or “absent” online by the deadline. Results submitted after the deadline are not graded. Participating laboratories are required to indicate the test methodology on the result form: antibody-based flow cytometry, micro-cytotoxicity, molecular methods: PCR-SSO, PCR-SSP, and real-time PCR. An “other test methodology category” is provided for methods that do not fall under the listed methods. Exception codes can be used if a laboratory cannot perform the PT. The survey occasionally includes some dry challenge questions, which are educational and ungraded.

### 7.2 Unique considerations for proficiency testing for HLA-B27 typing

It is well known that HLA-B27 shows remarkable polymorphism, and its disease association varies in populations and with different alleles (Khan, 2017). HLA-B*27:05 is the most common disease-associated subtype in the world (Reveille, 2006a), whereas HLA-B*27:02 is more commonly seen in Mediterranean populations and HLA-B*27:04 in Asian populations. Other subtypes, namely, HLA-B*27:06 (a common subtype in Southeast Asia) and HLA-B*27:09 (a rare subtype found primarily on the Italian island of Sardinia), seem to lack the disease association (Reveille, 2006b). Thus, HLA-B27 allelic typing provides a better understanding of disease association. This is possible by high resolution typing methodologies like NGS. This PT survey is a screen for HLA-B27 and does not differentiate between the alleles. A review of responses to dry challenge questions demonstrates that most participants understand that allele-level HLA-B27 typing results can inform clinical interpretation and how they impact the risk association (Pena et al., 2023).

### 7.3 Grading proficiency testing for HLA-B27 typing

Results are provided by test methodology for laboratories to evaluate their performance in their “methodology” peer group. However, grading is performed by consensus of >90% and not by methodology. The CAP uses exception reason codes for ungraded results. The laboratory must identify all analytes with an exception reason code, review, and document the acceptability of performance as outlined in the instructions and retain documentation of review for at least 2 years. Laboratories performing B27 testing by flow cytometry may need additional confirmation by an alternate method and may send a patient specimen to another center for additional testing. Since this is not permitted for PT specimens, these laboratories must report their flow cytometry result and indicate that they would normally send such a patient sample for additional testing. Laboratories with the indeterminate result indicating that they would send this patient specimen out for additional testing are graded acceptable.

### 7.4 Future of proficiency testing for HLA-B27 typing

Flow cytometry is the most used methodology due to its simplicity, cost-effectiveness, and fast turn-around time. However, it is also associated with higher error rates compared to all other methods due to cross-reacting antibodies and altered or masked antigenic epitopes (Kirveskari et al., 1997; Levering et al., 2003). There has been a gradual decrease in the number of laboratories performing flow cytometry methodology and an increase in molecular methods (Pena et al., 2023). Molecular typing would allow for a more accurate risk association and obviate the need for reflex confirmatory testing when “indeterminate results” are obtained by flow cytometry. The HITC may consider incorporating high-resolution typing results along with racial/ethnic data in future PT to aid in clinical interpretation and diagnostic classification.

## 8 Conclusion

Inaugurated seven and a half decades ago, the CAP PT program has not only withstood the test of time but has also evolved, mirroring an unwavering dedication to enhancing laboratory practices. This evolution is enabled by HITC providing subject expertise in the design and review of PT challenges that support the rapidly-evolving landscape of histocompatibility and identity testing.

The future trajectory of PT in these domains is aptly characterized as a dynamic and responsive continuum, inextricably aligned with the ever-evolving terrain of clinical diagnostics and patient welfare. The HITC, in recognition of its pivotal role, acknowledges the imperativeness of harmonizing with the needs of the HLA community. This entails catering to the specific lexicons and benchmarks delineated by organizations such as the National Marrow Donor Program (NMDP) and the United Network for Organ Sharing (UNOS). Furthermore, the committee anticipates an expansion of its PT purview to encompass burgeoning diagnostic modalities as well as the needs of international laboratories. For instance, on the horizon, donor-derived cell-free DNA is an emerging evaluative tool in the clinical assessment of solid organ transplantation rejection (Kant and Brennan, 2022). As laboratories incorporate this technology into their test menus, innovative PT will be needed to ensure quality and patient safety. In the domain of customer service, the committee recognizes the importance of expeditious and precise responses to participant queries, which will be aided by integration of enhanced bioinformatics and data analytics software. Through the implementation of continuous improvement measures, the committee aims to remain proactive and responsive to the challenges arising from the ongoing advancement of HLA antibody testing and molecular typing.

In summation, the HITC hopes the contents of this review serve as a comprehensive resource, shedding light on the rich history and promising future of the CAP PT program, a cornerstone of laboratory quality assurance and proficiency assessment in the field of histocompatibility and identity testing. It amplifies the CAP’s enduring pledge to excellence and commitment to continuous improvement, ensuring that laboratories across the country and beyond maintain the highest standards in patient care and clinical testing.
